# Polymorphism of the CYP2C9 and VKORC1 genes in patients on the public health system of a municipality in Southern Brazil

**DOI:** 10.1590/1677-5449.200214

**Published:** 2021-05-19

**Authors:** Christiane Colet, Mariana Rodrigues Botton, Karin Hepp Schwambach, Tânia Alves Amador, Isabela Heineck

**Affiliations:** 1 Universidade Federal do Rio Grande do Sul – UFRGS, Porto Alegre, RS, Brasil.; 2 Universidade Regional do Noroeste do Estado do Rio Grande do Sul – UNIJUÍ, Ijuí, RS, Brasil.; 3 Hospital de Clínicas de Porto Alegre, Porto Alegre, RS, Brasil.; 4 Universidade Federal de Ciências da Saúde de Porto Alegre (UFCSPA), Porto Alegre, RS, Brasil.

**Keywords:** warfarin, genetic polymorphism, adverse effects, varfarina, polimorfismo genético, efeitos adversos

## Abstract

**Background:**

Genetic factors can be responsible for part of the populational and interindividual differences observed in warfarin users.

**Objectives:**

To identify occurrence of polymorphisms of the CYP2C9 and VKORC1 genes in patients taking warfarin and relate these profiles to their medication dosages and the Time in Therapeutic Range (TTR).

**Methods:**

Monthly interviews were conducted for data collection. Data were collected on demographic characteristics and medications in use, especially warfarin, including reason for prescription and weekly dose. TTR was calculated as the percentage of days with international normalized ratio (INR) between 2 and 3. The CYP2C9 and VKORC1 genes were analyzed at a Human Genetics Laboratory.

**Results:**

49 patients (74.2%) had polymorphisms of the CYP2C9 and/or VKORC1 genes; the remaining 17 (25.8%) did not have these polymorphisms. The average weekly dose of warfarin was lower among those who had a polymorphism for any of the genes compared to those who did not, with a significant difference (p = 0.035). The mean TTR was also lower among patients with polymorphism. However, the difference between the two groups was not significant for this variable (p = 0.438).

**Conclusions:**

An association was observed between the polymorphisms and the warfarin doses taken by the patients. However, there was no association with adverse events or the time spent within the therapeutic range in this sample.

## Resumo

**Contexto:**

Fatores genéticos podem ser responsáveis por parte das diferenças populacionais e interindividuais observadas em usuários de varfarina.

**Objetivos:**

Identificar a ocorrência de polimorfismo dos genes CYP2C9 e VKORC1 em pacientes em uso de varfarina e relacionar esses perfis com a dose do medicamento e o tempo no intervalo terapêutico.

**Métodos:**

Foram realizadas entrevistas mensais para a coleta de dados. Foram reunidos dados sobre características demográficas e medicamentos em uso, principalmente sobre varfarina, como motivo da prescrição e dose semanal. O tempo no intervalo terapêutico foi calculado como a porcentagem de dias com razão normalizada internacional entre os valores 2 e 3. Os genes CYP2C9 e VKORC1 foram analisados em laboratório de Genética Humana.

**Resultados:**

Entre os participantes, 49 pacientes (74,2%) apresentaram polimorfismo dos genes CYP2C9 e/ou VKORC1; os 17 restantes (25,8%) não apresentaram esses polimorfismos. A dose média semanal de varfarina foi menor entre os que apresentaram polimorfismo para algum dos genes em comparação aos que não apresentaram, com diferença significativa (p = 0,035). O tempo no intervalo terapêutico médio também foi menor entre os pacientes com polimorfismo. Porém, não houve diferença significativa entre os dois grupos para essa variável (p = 0,438).

**Conclusões:**

Foi observada associação entre os polimorfismos e a dose de varfarina utilizada pelos pacientes; no entanto, não houve associação com eventos adversos e o tempo de permanência na faixa terapêutica nessa amostra.

## INTRODUCTION

Several factors can modify the anticoagulant effect of warfarin. Of these, genetic factors are responsible for part of the populational and interindividual differences observed among warfarin users.^1^

In conjunction with environmental factors, the CYP2C9 and VKORC1 genotypes explain two-thirds of inter-individual variability in response to warfarin. The VKORC1 and cytochrome P450 (CYP) 2C9 genes affect the pharmacodynamics and pharmacokinetics of warfarin, respectively.^2^

Identification of polymorphisms in patients is important for establishing the appropriate dose of warfarin and for preventing adverse events, especially at the beginning of treatment.^3^^,^^4^ However, these tests are not yet available on the Brazilian public health system (known as the SUS), because of their high cost. Brazil is a country of continental proportions with an ethnically diverse population and the genetic profile of its population can therefore exhibit great variability. This study aims to identify the occurrence of polymorphisms of the CYP2C9 and VKORC1 genes in patients taking warfarin in a municipality in southern Brazil and to relate these profiles with drug dosage and Time in Therapeutic Range (TTR).

## METHODS

The data used for this analysis are part of a prospective open cohort that includes warfarin users linked to the public health network in the municipality of Ijuí, RS, Brazil, which has a population of 79,396 inhabitants. Data related to drug interactions and adverse events^5^ have been published and data on patients’ therapeutic itineraries have also been published.^6^

### Study participants, data collection and organization

The Municipal Pharmaceutical Services offers 15 sites where drugs can be dispensed, linked to Basic Health Units (BHU). The municipality studied does not have a computerized system for dispensing drugs and copies of prescriptions are stored at the Municipal Secretariat of Health (SMS) Central Pharmacy.

The sample calculation considered the total population of the municipality, a 90% confidence level and 10% error, resulting in 69 subjects. The sample was selected by convenience. All prescriptions that contained warfarin were identified and selected and the patients’ charts were accessed, enabling identification of residential addresses, telephone data, and, thus, scheduling of home visits. We included all patients taking warfarin orally, for chronic treatment, living in the city of Ijuí, RS, who were outcome-free at baseline. Participants were identified in February 2014 and monitored for a maximum period of 18 months and a minimum of 12 months, between April 2014 and October 2015. New patients were recruited at the beginning of their warfarin treatment for the first six months of the study.

Data collection comprised monthly interviews conducted at the participants’ homes. Demographics data such as gender and skin color were collected in addition to specific data on the drugs being used – especially warfarin – such as reason for prescription and weekly dose. Adverse events like bleeding, thrombosis, hospitalizations, and deaths were recorded. These outcomes were self-reported and confirmed in medical records for cases in which the user sought medical care on the municipal care network. Details on patient identification, patient inclusion and information collection are described in Colet et al.^6^

Samples were also collected for laboratory tests at the participants’ homes in the third, tenth and, eighteenth months of the study. The results of INR (international normalized ratio) tests were used to calculate the time that patients taking warfarin remained in the therapeutic range (TTR), employing validated methodology adapted by Schmidt, Speckman, and Ansell.^7^ The TTR was calculated as the percentage of days with INR between 2 and 3. The median and mean TTR values were used to present these data. The molecular analysis was performed at the Human Genetics Laboratory of the Department of Genetics at the UFRGS. For the analysis of the CYP2C9 and VKORC1 genes, 5 mL of venous blood was collected in 3.2% sodium citrate. DNA extraction was performed using Invitrogen’s commercial PureLink® DNA extraction kit. The identification of the -1639G> A (rs9923231) polymorphisms in the VKORC1, CYP2C9*2 (rs1799853) and CYP2C9*3 (rs1057910) gene, in the CYP2C9 gene, was responsible for the real-time PCR technique. Probes synthesized and standardized by the company Life Technologies® were used, specific for each polymorphism.

### Statistical methods

Data were analyzed using SPSS version 18. Descriptive statistics of central tendency and dispersion were calculated. The magnitudes of associations were determined using Fischer’s exact test and one-way ANOVA was used to assess the associations between dose and polymorphism. The Chi-square test was used to assess associations between polymorphism, dose, and ethnicity.

### Ethical aspects

All research participants signed an Informed Consent Form. The project was approved by the Research Ethics Committee at the Universidade Federal do Rio Grande do Sul (UFRGS), under number 336.259/2013.

## RESULTS

This study evaluated the data of 66 patients from the original cohort, composed of 69 warfarin users – three patients did not take the molecular tests. Most patients were female (35; 53.0%) and white (55; 83.3%). Mean age was 64.3 ± 13.7 years, with a minimum of 33 and maximum of 96 years. The complete sociodemographic data are described in Colet, Amador, and Heineck.^6^

**Figure 1:**
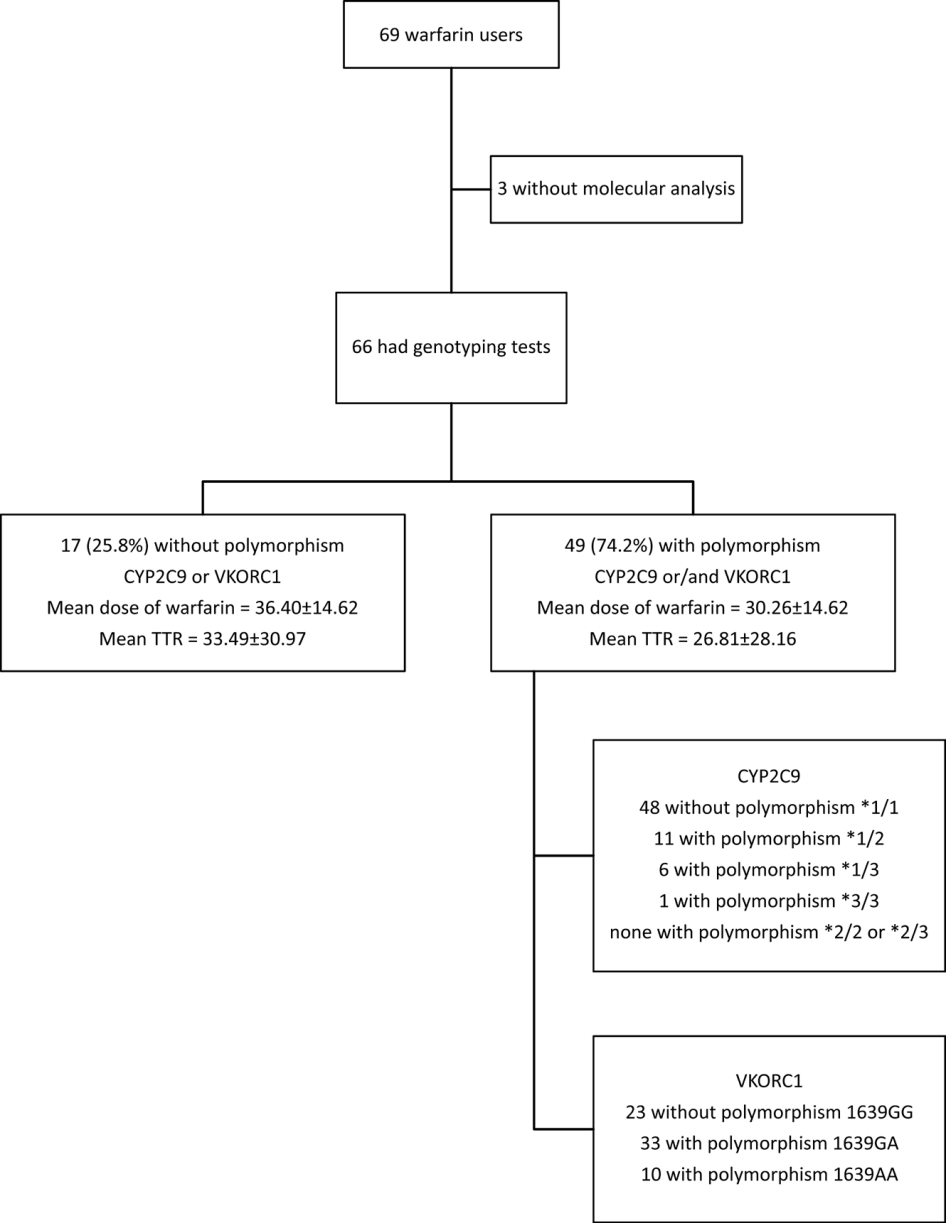
Flowchart illustrating polymorphism analyses for the CYP2C9 and VKORC1 genes, average dose of warfarin, and average TTR in a cohort of patients from the municipality of Ijuí, RS, Brazil (n = 66).

Participants used an average of 10.5 ± 3.4 continuous use drugs, including warfarin. The most frequent reason for warfarin use was prosthetic heart valves (39.7%), followed by treatment or prevention of venous thromboembolism (36.3%). Forty-nine patients (74.2%) had polymorphisms of the CYP2C9 and/or VKORC1 genes; the remaining 17 (25.8%) did not have these polymorphisms (Figure 1). There were no associations between polymorphism and sex (p = 0.986) or skin color (p = 0.304).

According to Figure 1, we can see that the mean weekly warfarin dose was lower (30.26 ± 14.62) among those who had polymorphisms of any of the genes, compared to those who did not (36.4 ± 13.9), with a significant difference (*p* = 0.035). Mean TTR was also lower among patients with polymorphisms. However, there was no significant difference between the two groups for this variable (*p* = 0.438).

Table 1 shows data on the mean weekly warfarin dose and the mean TTR according to the genotypes observed. No patient had the genotypes CYP2C9 *2/2 or CYP2C9 *2/3. Evaluating each genotype, it was found that those without polymorphism of the CYP2C9 gene (* 1/1) were taking higher doses than those who had polymorphisms of this gene (* 1/2, * 1/3, * 3/3), with significant difference (*p* = 0.013). Likewise, for the VKORC1 gene, there was a significant difference in dose between the different genotypes (*p* = 0.018). On average, patients with the CYP2C9*1/1 genotype remained less time in the therapeutic range than those with polymorphisms of this gene; but no significant association was observed between mean TTR and these different polymorphisms (*p* = 0.656). The analysis based on median TTR, calculated at 28.6%, allowed us to observe that 24 of the 47 patients with a CYP2C9 * 1/1 profile remained above the median 75% of the time, showing better performance than the other profiles; this difference was not significant, however (*p* = 0.193). Regarding the VKORC1 gene, there was also no significant difference between the groups considering mean TTR (*p* = 0.450) or median TTR (*p* = 1.000). There were no significant differences in relation to the different genotypes in terms of the adverse events bleeding (*p* = 0.613), thrombosis (*p* = 0.428), or hospitalizations (*p* = 0.075).

**Table 1:** Associations between weekly dose and TTR with CYP2C9 and VKORC1 genotypes among warfarin users in a cohort of patients in the municipality Ijuí, RS, Brazil (n=66).

Genotype	CYP2C9				*p*	VKORC1			*p*
	*1/1	*1/2	*1/3	*3/3		1639GG	1639GA	1639AA	
N	48	11	6	1		23	33	10	
Weekly dose (mg) (Mean ± SD)	27.2 ±18.1	16.4 ±12.3	18.3 ±10.7	8.8	0.013*	27.7 ±16.8	23.2 ±18.8	20.5 ±10.9	0.018^*^
TTR (Mean ± SD)	27.8 ±23.4	31.4 ±34.5	33.0 ±28.4	0.0	0.656	31.3 ±27.1	25.5 ±24.9	33.9 ±12.9	0.450
Median (28.6%)									
Below (n; %)	23 (67.6)	5 (14.7)	6 (17.6)	0	0.193	12 (35.3)	17 (50.0)	5 (14.7)	1.000
Above (n; %)	24 (75.0)	6 (18.8)	1 (3.1)	1 (3.1)		12 (37.5)	15 (46.9)	5 (15.6)	

* p-value < 0.05.

## DISCUSSION

In this study, it was observed that patients with polymorphism of the CYP2C9 and/or VKORC1 genes took significantly lower warfarin doses than those without polymorphisms. The same results were found in other studies.^3^^,^^7^^-^9 In the study by Botton et al.,^4^ polymorphisms of the CYP2C9 gene were strongly associated with dosage in individuals with genotypes CYP2C9 *1/*2, CYP2C9 *2/* 2, CYP2C9 *1/*3, and CYP2C9*2/*3, who required 17.8%, 59.6%, 26.5%, and 52.9% lower doses respectively, compared to homozygotes for the wild type genotype. Other authors corroborate that the initial warfarin dose should be 5 mg/day, adjusted by the INR value and the underlying disease to indicate the use of anticoagulants.^10^

Mean TTR was also lower among patients with polymorphisms, without significant difference, however. Another finding is that data from the present study shows that only a quarter of the interviewees did not present any polymorphism for one or both of the genotypes surveyed. Botton et al.^4^ observed similar results. These data demonstrate that most of the patients monitored have a polymorphism in the genes investigated and, therefore, need dose adjustments and greater monitoring to avoid adverse events.

Perini et al.^9^ argue that ethnicity is related to warfarin dose, reporting that white patients had an average dose of 28.9 mg ± 12.3 (± SD, n = 196), brown patients of 32.9 mg ± 12.4 (n = 118), and black patients of 35.3 mg ± 14.6 (n = 76). In the case of the Brazilian population, one must consider its high ethnicity diversity, which can influence the frequency of polymorphisms. The low frequency of black and brown patients in the population of this study impaired the analysis of polymorphism in relation to ethnicity. Suarez-Kurtz and Botton^11^ conducted a review study with patients of African descent, finding that there are polymorphisms with a high frequency in this population that rarely appear in individuals of European descent. Thus, black and Latino populations have greater variability in warfarin doses and are at greater risk of suffering warfarin-related adverse events compared to those of European descent.^2^

Carriers of the CYP2C9* 2 and/or the CYP2C9*3 allele, which account for 10% and 6% of the European population, respectively, are at increased risk of bleeding complications, especially at the beginning of anticoagulant therapy and need more time to stabilize the dose of warfarin. According to Mandic et al.,^12^ the risk of bleeding complications during warfarin treatment increases by 90% for the CYP2C9 *2 allele and by 80% for the CYP2C9 *3 variants.

In the present study, it was found that patients’ TTR was low and that they remained about 70% of the time outside the target INR, staying outside the therapeutic range for longer. Although no significant association was observed between median TTR and the polymorphisms analyzed, this data may be a consequence of either sample size or chronic patients, since another study has already shown this association.^13^ In a cohort study conducted in Canada with 1059 patients taking warfarin, the average time in the therapeutic interval was 56% (± 25%) in the three months after start of treatment and 70% (± 21%) in the 3 to 12 months interval. Independent predictors of inadequate anticoagulation control were chronic kidney disease, heart failure, dyslipidemia and age.^14^

The CYP2C9 and VKORC1 genotypes are among the main determinants of warfarin dose, as well as of the risks for excess anticoagulation and hemorrhage, especially in the initial months of therapy.^9^ In this study, polymorphisms were not associated with bleeding; however, the patients monitored were not aware of their genetic profile and most were chronic warfarin users. Several dose adjustments were likely made on the basis of INR test results due to adverse events at the beginning of treatment.

The present study had limitations. The sample size calculated was achieved, but three subjects were lost to the analysis. The sample was considered too small for other statistical inferences and subgroup analyses. A detailed analysis of potential drug interactions was not performed, nor was an analysis of the influence of the underlying disease on clinical outcomes. On the other hand, the collection of material and monitoring of warfarin users in a local setting can be considered a strong point of this study. These data can contribute to rational and safe use of this drug.

The INR test for follow-up can be performed in outpatient clinics or by the patients themselves at home. In this study, patients performed the tests in the laboratory upon medical request and were followed-up at a Basic Health Unit. In these settings and also in specialized clinics, genetic selection can help to identify patients who need doses different from those recommended for the general population.^15^

Knowledge of the pharmacogenetics of coumarins can help the health team to adjust each patient’s therapeutic dose, thus reducing the risk of bleeding at the beginning of treatment and over the course of treatment with anticoagulants.^16^ Genotype-guided warfarin dosing algorithms are a rational approach for optimizing warfarin dosing and potentially reducing adverse events, but improvements to warfarin dosing algorithms between racial/ethnic groups will be necessary for successful clinical implementation of the pharmacogenomics of warfarin.^2^

## CONCLUSION

This study observed an association between these patients’ polymorphisms and the warfarin doses they were taking, although there was no association with adverse events or with the time spent in the therapeutic range in this sample size.

Genetic polymorphism testing of anticoagulated patients is not yet available for Brazilian patients using the public health system and they are only conducted as part of research, as in the present study. There was a high frequency of patients with genetic polymorphisms and, therefore, different responses to use of anticoagulants. Additional studies with larger numbers of patients and cost-benefit studies are needed to justify routine use of these tests in our population.
